# Mr. Hey, on the Fungus Hœmatodes

**Published:** 1804-03-01

**Authors:** William Hey

**Affiliations:** Leeds


### *To the Editors of the Medical and Physical Journal.*

Gentlemen,

THE discussions which have taken place respecting that species of tumour, mentioned in my volume of *Practical Observations* lately published, to which I have given the name *fungus hæmatodes*, seem to call for some attention on my part; as it is of consequence to mankind that diseases should be accurately known, and carefully distinguished.

Your valuable correspondent, Mr. Simmons, of Manchester, is of opinion, (see Journal for July 1803) that the disease is a spurious aneurysm; while another gentleman of great experience, who takes much interest in the newly established Institution for the investigation and cure of cancerous diseases, is pleased to consider the fungus hæmatodes as a species of cancer.

A difference so great in the ideas of medical men, respecting the disease in question, will naturally excite a suspicion, that there is something obscure in the nature of the the complaint, or in the description given of it. The subject may, therefore, merit a little farther investigation.

Before I proceed to examine the opinion of Mr. Simmons, I must observe, that he considers the disease of the knee-joint, described by Mr. Russell of Edinburgh, and called by him "an uncommon disease," as the same which I have described under the term of *fungus hæmatodes*. (See Med. & Phys. Journ. for July 1803, p. 2.) But whoever will carefully compare our descriptions must see a considerable difference between them.

1. Mr. Russell considers the disease, which he describes, as peculiarly affecting the knee-joint; whereas I have never yet seen the fungus hæmatodes affecting that joint, though I have seen it in most parts of the human body. Although the tumour in Case 1,* began on the side of the knee near the patella, and remained fixed in that part above half a year, before it extended up the thigh; yet, after amputation, "the joint of the knee was found to be perfectly sound." †

2. Mr Russell describes the substance forming the tumour in his cases as "almost transparent and colourless, and a soft gelatinous consistence." p. 75. Whereas the fungus hæmatodes has always, after amputation, been found by me quite opake, "of a variegated reddish colour" mixed with white or grey, and of a greasy or unctuous nature, "nearly resembling the medullary part of the brain." p. 237.

3. In Mr. Russell's cases, "every patient," (except one, whose disease was somewhat different from the rest) "on whom amputation was performed, died from the hæmorrhage—which invariably occurred in every case of the disease, and at very different periods from the date of the operation." p. 77. On the contrary, all the patients, upon whom I have seen amputation of an extremity performed for the fungus hæmatodes, have recovered without any disposition to hæmorrhage subsequent to the operation.

These circumstances certainly point out a material difference in the nature of the diseases described by Mr. Russell and myself; I shall, therefore, confine the following remarks to the cases which I have related, and proceed to shew, that several of them bore no affinity to the spurious aneurysm, nor to cancer.

In performing the operation of amputation in Campinet's

* Practical Observations, p. 233. † Ibid. p. 239.

net's case, (Case 1, p. 231) I left a small superficial part of the sac, near the extremity of the stump, not doubting that this wound in the integuments would soon granulate, and heal together with the extremity of the stump. No surgeon, I suppose, can imagine that this superficial sore could cause a spurious aneurysm. "The granulations of flesh upon the stump became good, and the progress of healing was favourable." Nay, "that small and superficial part of the great sac—was healed;" yet a tumour rose beneath the cicatrix, containing a soft substance, like the brain, which formed for itself cells in the surrounding parts. Two attempts to extirpate this substance proved fruitless, so that I was obliged to amputate a second time, when I found the femoral artery not "incontracted," but impervious.

In Mrs. Storr's case (Case 4, p. 264), I extirpated a tumour in the mamma, and "about six weeks after the complete cicatrization of the wound, Mrs. S. began to feel a constant uneasiness in the part, and perceived it to be tumefied." Has such a disease as this any affinity with the spurious aneurysm? Yet this tumour was formed by a mass of unctuous matter, similar to that described in the first case.

Again, I extirpated the whole of this mass, with the assistance of Mr. Lucas," so that "no part of the integuments was left that had the least morbid appearance; and the disease seemed to be completely removed." p. 266. "The wound was soon filled with good granulations, and the cure proceeded in the most favourable manner for three weeks." Surely Mr. Simmons will not say, that there was any circumstance in this case that could give rise to a spurious aneurysm? The whole case completely excludes any such idea. Let any experienced surgeon read this case with attention, and he must acknowledge, that the morbid fungus, repeatedly formed, and repeatedly removed, can never with any propriety of language be classed under the head of spurious aneurysm.

The same reasoning will apply to such cases as that of James Richardson, where the tumour was situated "on the posterior part of the shoulder,"—"between the integuments and external muscles," (Case 9, p. 277, 278.) and "had arisen to a considerable size before the patient was aware of its existence." He remembered no circumstance that could have given rise to this tumour. He died about ten months after I last saw him. His widow informed me lately, that a substance broke through the skin about ten weeks weeks before his death, and grew to the size of a man's head. It bled frequently, as if blood had been squeezed from a sponge.

Mr. Simmons supposes that I, and others who saw the operations which I have described, were deceived in thinking the morbid masses to be organized, and declares, "that he cannot yield assent to the existence of it" (organization) "until injections have fully established the fact."— He imagines, that upon our handling the masses, we only made way for the flux of blood recently effused from the ruptured arteries. But Mr. S. in this objection, overlooks the fact related by me, that in several of the cases blood issued from the masses when they were not handled. In Mr. Ward's case (Case 6.) the fungus bled considerably after it had been punctured with a lancet by an old woman, under whose care he had placed himself, p. 271. The delay of a week in performing the operation, which arose chiefly from his own reluctance, was the cause of his being "much reduced by the loss of blood from the fungus," p. 271, though his leg was kept still in a horizontal position during the greatest part of this interval.

In the case of Ann Wood, (Case 10.) who was Mr. Logan's patient, a hæmorrhage took place from one of the excoriated parts" of the fungus, "three months before her admission" into the Infirmary, "at which time she lost about eight ounces of blood, p. 280. The fungus bled repeatedly, though in small quantities after the young woman was taken into the house. If Mr. S. had been present when Mr. Logan and I saw blood issuing from the excoriated parts of the tumour, I suppose he would have thought the hæmorrhage before amputation as good a proof of the organization of the mass, as an injection after the operation.

As Mr. S. seems disposed to deny the existence of the fungus hæmatodes, as a disease sui generis, I rather wonder that he takes no notice of Mr. Burn's dissertation on *Spongoid Inflammation.* This disease agrees in so many particulars with the fungus hæmatodes, that I am inclined to consider them as one, though Mr. B.'s description does not intirely coincide with my own. He mentions the enlargement of the lymphatic glands as a common occurrence in the disease, and as rendering the amputation of an extremity unsuccessful, unless performed at an early period. Whereas in five cases of the fungus hæmatodes on the extremities, in which amputation was performed after the disease had subsisted a considerable time, no enlargement of the lymphatic glands was perceived, nor did any sub-

 sequent sequent affection of these glands render the operation abortive.

As the fungus hæmatodes is often formed under circumstances which exclude the idea of a spurious aneurysm; so also is it distinguishable from a cancerous affection.

This usually, I might perhaps say always, begins with some degree of schirrous hardness in the part affected. Such induration takes place not only where the glands are the seat of the disorder, but also in cancerous affections of the viscera and common integuments. The uterus first becomes schirrous, and the vagina is beset with tubercles before ulceration commences. Whereas the fungus hæmatodes feels soft, spongy, and elastic, though unequally so. While it remains covered with the common integuments, it often gives the sensation in some parts, when handled, as if some fluid lay deep seated within it, while other parts feel as if there was a thickening of the common coverings of the body.

When a cancerous tumour becomes ulcerated, it throws out an excrescence somewhat resembling the head of a cauliflower. But I have seen the fungus hæmatadoes burst through the skin without the slightest induration of the surrounding integuments. The fungus did not then appear like a cancerous excrescence, but rose with a dark coloured surface somewhat resembling coagulated blood, yet apt to bleed freely.

The fungus which repeatedly arose in Mrs. Storr's breast (see Case 4.) after the wound had been cicatrized, or filled with good granulations, had not at all the cancerous aspect. It was soft, of a greasy consistence, and was not surrounded with an indurated margin.

Such cases as that of Ann Wood, (Case 10.) bear the greatest resemblance to cancer; but they differ in their origin, and in the texture of the mass, when divided after amputation, which shews nothing schirrous, but resembles the brain in consistence; and with respect to its colour, has a variegated appearance.

The case of Mr. Ward (Case 6.), to which I have already referred, shews a manifest difference both from spurious aneurysm and cancer.—But I will not enlarge on this subject—The cases and observations which I have already published are faithful copies from nature; and a little time will, I doubt not, convince the judicious practitioner, that there is a disease, of a peculiar nature, to which I have given the name of FUNGUS HÆMATADOES, which has not been received into the list of diseases by the most eminent nosologists, nosologists. A disease which proves certainly fatal when left to itself, and for the cure of which no remedy has been hitherto discovered except amputation of the limb, when it has happened to be seated on one of the extremities of the body.*

*Leeds,*
*Jan.* 27, 1804.

I am, &c.

WILLIAM HEY.

* Under some favourable circumstances a tumour of this kind might probably be extirpated from some other part, if an opportunity was afforded before the fungus had sunk deep, and formed pouches in the surrounding soft parts.

